# Serum and gene expression profile of cytokines following combination of yoga training and vitamin D supplementation in breast cancer survivors: a randomized controlled trial

**DOI:** 10.1186/s12905-022-01671-8

**Published:** 2022-03-24

**Authors:** Mahdi Naderi, Hajar Kordestani, Zahra Sahebi, Vahid Khedmati Zare, Sadegh Amani-Shalamzari, Mojtaba Kaviani, Joachim Wiskemann, Mahdieh Molanouri Shamsi

**Affiliations:** 1 Departement of Exercise Physiology, Faculty of Physical Education and Sports Sciences, Kharazmi University, Tehran, Iran; 2 Department of Exercise Physiology, Faculty of Physical Education and Sports Science, Kharazmi University, Tehran, Iran; 3 School of Nutrition and Dietetics, Faculty of Pure and Applied Science, Acadia University, Wolfville, NS Canada; 4 Working Group Exercise Oncology, Department of Medical Oncology, National Center for Tumor Diseases (NCT), Heidelberg University Hospital, Heidelberg, Germany; 5 Department of Physical Education and Sport Sciences, Faculty of Humanities, Tarbiat Modares University, Tehran, Iran

**Keywords:** Hatha yoga, Handgrip strength, Quality of life, Pro-inflammatory cytokines

## Abstract

**Background:**

This study aimed to examine the effect of the combination of yoga training with high vitamin D dose supplementation on the expression and systemic levels of inflammatory cytokines and psychophysical status of breast cancer survivors.

**Methods:**

Thirty volunteered breast cancer survivors (48 ± 8 years) were randomly allocated to a high dose (4000 IU) of vitamin D supplementation (HD) group (n = 10), yoga with a high dose of vitamin D (YHD) group (n = 10), and yoga with a low dose (2000 IU) of vitamin D (YLD) group (n = 10). Participants performed the Hatha yoga style for 12 weeks, twice a week. Blood samples, quality of life (QoL) questionnaire, and physical performance tests were taken before and after the intervention.

**Results:**

Body fat percentage (*ηp*^2^ = 0.36), handgrip strength (*ηp*^2^ = 0.41) and QoL indicators include global health (*ηp*^2^ = 0.54), functional scales (*ηp*^2^ = 0.49), and symptoms scales (*ηp*^2^ = 0.50) were significantly improved in the both YHD and YLD groups compared to the HD group (*p* < 0.05). Also, interleukin-10 (IL-10) levels were markedly increased in the Y-HVD group compared to the Y-LVD and HVD groups. Moreover, there were significant decreases in tumor necrosis factor-α (TNF-α) and interleukin-6 levels in the Y-HVD group after the intervention. The anti-inflammatory index (IL-10/TNF-α) was significantly increased in both the yoga groups (*P* < 0.05).

**Conclusion:**

Yoga promotes physical and psychological fitness and, in combination with a high dose of vitamin D, improves the cytokine profile, which can effectively manage the side effects associated with cancer.

*Trial registration* IRCT20210726051993N2. Registration date: 2022/02/27. URL: https://www.irct.ir/trial/62079

## Background

Breast cancer (BC) and treatments have several side effects on psychological and physical health, leading to a decline in the quality of life (QoL). The side effects could reduce survivors' muscle strength and aerobic capacity [1] and expose patients to psychiatric disorders such as depression and anxiety [2]. Fatigue is a common side effect of cancer treatment associated with decreased physical activity, which subsequently reduces functional abilities.

There are several lines of evidence linking fatigue to elevated pro-inflammatory cytokines in cancer survivors [3, 4]. In particular, serum levels of interleukin-6 (IL-6) [5] and tumor necrosis factor-α (TNF-α) [6] may be increased as part of the host response to tissue damage or cancer treatments. Even though IL-10 is considered an anti-inflammatory cytokine, a dual role of IL-10 in breast cancer development was reported [7]. The IL-10/TNF-α ratio is widely used as an anti-inflammatory status [8] and metabolic diseases [9]; thus, this ratio would be a better indicator of the treatment process. However, peripheral blood mononuclear cells (PBMCs) are considered the primary source of pre-and anti-inflammatory cytokines; they are involved in changes in serum cytokine profiles [10]. It appears that investigating inflammatory markers-related gene expression in PBMCs may predict their changes in different cancer stages more accurately [11]. It is suggested that behavioral modifications such as exercise and a healthy diet effectively regulate cytokine balance and manage adverse side effects [12, 13].

Many BC patients and survivors, especially at menopause, have deficiencies in nutritional indicators, including vitamin D (VD) [14, 15]. Vitamin D deficiency is associated with a decline in QoL [16], worsens cancer prognosis, and increases the mortality rate in cancer patients [14, 17]. However, more studies are needed on the VD doses that can be used to improve the QoL, especially in cancer survivors. Immune regulatory effects of VD supplementation, particularly in high doses was observed in some studies [18, 19]. Mechanistically, VD supplementation could lead to a shift from a Th1 to a Th2 phenotype [20], and enhance IL-10 gene expression in T cells [21], which in turn inhibits the production of pro-inflammatory cytokines.

Exercise training is a behavioral modification to alleviate the side effects of cancer and its treatments. Yoga is a body-mind exercise that combines physical, mental, and spirit to improve psychological and physical health. Hatha yoga, the most common style executed in therapeutic settings, includes physical exercises (Asanas), breathing techniques (Pranayama), and meditation (Dyana) [22, 23]. A systematic review reported that chronic stress via dysfunction in the classic neuroendocrine system and the sympathetic nervous system could induce tumorigenesis and promote cancer development [24]. Thus, stress management is crucial for cancer patients. Although the results are contradictory, it has been shown that yoga dampens inflammatory markers [25], stress and anxiety [26, 27], and fatigue [28] in cancer survivors; hence, yoga appears to be an appropriate approach to improving the QoL of women with breast cancer. Furthermore, some studies showed that yoga could have a regulatory effect on the nervous system [23]. Declined sympathetic nervous system tone [23, 29], and increased vagal activity [29] are of mechanistic factors observed in yoga’s benefits, both of which are involved in favorable endocrine and immune system changes that could lower inflammation markers. Therefore, in addition to improving physical and psychological fitness, yoga therapies could reduce inflammatory responses [25].

A combination of exercise training and antioxidant supplementation in high doses can be effective on anti-tumor immunology and inflammatory cytokines [30]. However, considering the immune system's changes, the simultaneous effect of exercise training and VD intake has not been fully elucidated. Overall, given that a high dose of VD is safe for women at higher risk of BC [31] and recommended for BC survivors to reduce inflammatory markers, yoga also plays an influential role in this process; we hypothesized that combining yoga exercise training and high VD dose can be more effective on expression and systemic levels of inflammatory cytokines. For approving our hypothesis, the effects of the combination of low VD dose and yoga exercise training on inflammatory cytokines were assessed in the current study. Moreover, inflammatory responses were associated with the psychological situation of breast cancer. Here, we assessed the possible relation between psychological indices and inflammatory expression in PBMCs and systemic levels of inflammatory cytokines in BC survivors.

## Methods

### The experimental approach to the problem

**Fig. 1:**
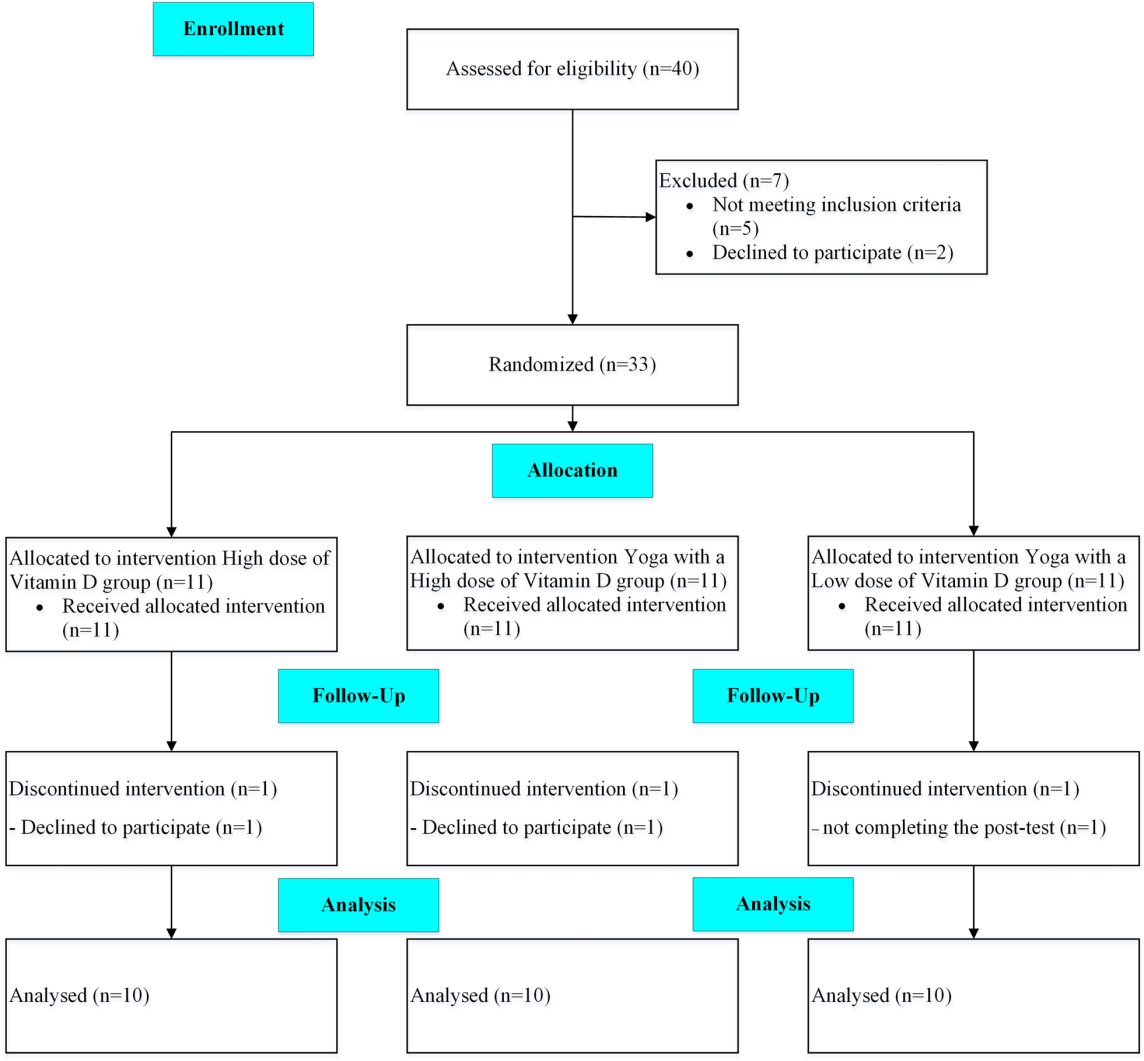
Schematic overview of study timeline (CONSORT flow diagram)

This randomized controlled trial, single-blinded, and parallel groups aimed to determine the effectiveness of 12 weeks of yoga training combined with VD supplementation on QoL and inflammatory markers in BC survivors. A few oncologists introduced eligible participants to participate in the study. Initially, based on the initial level of VD, the participants were divided into three groups randomly by a third person who was not in the research group. Pre- and post-intervention, QoL questioner, and handgrip strength tests were performed by the third assessor. Venous blood samples were collected to measure IL-6, IL-10, and TNF-α and their gene expressions in leukocytes. A schematic overview of the study is presented in Fig. 1.

### Participants

Following an initial meeting to discuss the objectives of this research project with oncologists, they referred the participants to the research team. Inclusion criteria were described in our previous study [32]. The history of BC survivors is presented in Table 1. Briefly, the tumor type of survivors was carcinoma in situ (stage 0), IA& IB stage, and IIA based on the TNM staging system. The sample size was calculated by using G*Power Software as described in our previous study [32]. Thirty-three BC survivors who met inclusion criteria volunteered to participate in the study, but the data of 30 participants (age: 47.90 ± 7.95 years; height: 160.93 ± 6.12 cm, body mass: 72.62 ± 11.72 kg) were obtained and analyzed finally. Three participants were excluded from the study as described in Fig. 1 and our previous study [32]. A third person randomly divided participants into three groups based on a simple block random method (6 blocks), including a high dose (4000 IU) of VD supplementation (HVD) group (n = 10), yoga with a high dose (4000 IU) of VD (Y-HVD) group (n = 10), and yoga with a low dose (2000 IU) of VD (Y-LVD) group (n = 10). It seems that we needed a group that only practices yoga, but all cancer survivors consume different doses of VD, so due to ethical reasons, we could not put that group.

**Table 1:** Survivor characteristics according to breast cancer subtypes

Group	Cancer type (TNM stage)			Type of treatment			Time after surgery (year)	Metastasis
	0	1	2	Chemotherapy (n)	Radio therapy (n)	Hormone Therapy (n)		
HVD	3	5	2	10	9	6	3.1 ± 0.8	No
Y-LVD	3	4	3	9	9	8	3.3 ± 0.8	No
Y-HVD	3	5	2	8	9	7	3.1 ± 0.9	No

*HVD* high dose of vitamin D, *Y-LVD* yoga with a low dose of vitamin D, *Y-HVD* yoga with a high dose of vitamin D, TNM staging system,*T* tumor size, *N* the number of lymph nodes involved, *M* metastases

## Measurements

### Physical measurements

A third person who was not a member of the research team conducted all measurements. Height, weight, and body fat percentage were measured as described in our previous research [32].

### Handgrip strength tests

A hand dynamometer with an adjustable grip (TKK 5101 Grip D; Takey, Tokyo, Japan) was used to assess handgrip strength. Participants performed two attempts with both hands, while arms were fully extended, forming an angle of 30° with respect to the trunk. The maximum score in kilograms for each hand was recorded, and the mean score of both hands was used in the statistical analyses.

### Quality of life

European Organization for Research and Treatment of Cancer Questionnaire (EORTC- QLQ-C30) was developed to assess the quality of life of cancer patients. The validity and reliability of this questionnaire were confirmed in the Iranian cancer population [33]. It consists of 30 questions that assess the global health, symptoms (fatigue, pain, nausea, and vomiting), and functional (physical, role, cognitive, emotional, and social) scales. Higher scores in global health and functional scales and a lower score in symptoms indicate better situations [34].

### Vitamin D supplementation

Participants received a dose of VD tablets according to our previous article [32]. Briefly, participants in the HVD and Y-HVD groups received VD tablets at 4000 IU daily, and individuals in the Y-LVD group received 2000 IU daily.

### Yoga protocol

A female certified yoga coach conducted yoga classes. Participants performed yoga according to our previous article [32]. Briefly, they performed yoga twice a week, for twelve weeks. Exercises were selected from the Hatha yoga style and included Asana (physical postures), pranayama (breath control), and Dyana (meditation). The yoga exercises begun with Pranayama (yoga mudra, Respiratory coordination), then asana (such as Marjaryasana cycle, Balasana, Hindolasana, Bhujangasana, Setu Bandha, Bitilasana, Surya namaskar, Baddha Konasana, Chakki Chalanasana, Utkatasana, Supta Baddha Konasana, Bhujangasana, kriya cycle, Salabhasana, Ardha Pavana Muktasana, Pavanamuktasana, suptaVakra Asana) and ended with dyana (Savasana). Each class lasted around 60–90 min; the yoga class started with 60 min and progressively increased 15 min each month to reach 90 min over the course. In order to monitor the intensity of yoga, the Borg Rating of Perceived Exertion (RPE) scale (6–20 score) was gathered after finishing every workout. The participants were asked to express their effort and exertion from the yoga practice. The Borg scale has a range from 6 to 20 (with 6 being no exertion at all and 20 being maximum effort). In general, the average score of the Borg scale was 8–14. The intensity of initial sessions was low (7–10 scores) and progressively reached moderate intensity towards (13–15) the end of the protocol.

### Cytokines assessments

A medical laboratory expert collected the blood samples (5 cc for separating serum, 2.5 cc in a tube containing Ethylenediaminetetraacetic acid (EDTA) for extracting PBMCs) in overnight fasting from an antecubital vein. Samples were spun at 3000 rpm in a 4 °C centrifuge for 10 min, and separated serum was stored a −20 °C. Specific human enzyme-linked immunosorbent assay [ELISA] kits were used to determine the serum level of IL-10 [catalog number: D1000b, R& D, US], TNF-*α* [catalog number DY210, R& D, US]*,* and IL-6 [catalog number D6050, R& D, US]. The intra- and inter-assay coefficients of variation were less than 8%. A buffy coat layer was removed using a suspension technic, and then RNA samples were extracted using the total RNA extraction Kit (NanoDrop Technologies/Thermo Scientific, Wilmington, DE, USA), and cDNA synthesis was performed using the Takara cDNA synthesis kit (Takara, Japan) according to the manufacturer's instructions. Real-time PCR was performed using the SYBR Green Master Mix kit (Ampliqon, Denmark). The thermal cycling program was described in our previous research [32]. GAPDH mRNA for the normalization of the gene expression analysis was used. The sequence of PCR primers used for the amplification of the protein-coding genes was as follow: IL-6 forward "GTGAGGAACAAGCCAGAGCA ", IL-6 reverse "TGGCATTTGTGGTTGGGTCA"; IL-10 forward “CTTTAAGGGTTACCTGGGTTGC", IL-10 reverse "CTCACTCATGGCTTTGTAGACAC"; TNF-α forward "CTCCCTCTCATCAGTTCCAT" and TNF-α reverse "CAGTTGGTTGTCTTTGAGATC"; GAPDH forward "CGAGATCCCTCCAAAATCAA" GAPDH reverse "AGGTCAGGTCCACCACTGAC". The fold change expression was calculated using the 2^−∆∆CT^ formula.

### Statistical analysis

Data were analyzed using SPSS software in accordance with the previous study [32]. Briefly, a paired t-test (within-group difference) and an analysis of covariance (ANCOVA) with Bonferroni post-hoc tests were used to analyze the effects of interventions on the variables. Data of gene expression changes were analyzed by ANOVA, and the magnitude and direction of the linear relationship between circulatory markers with anthropometric indicators and QoL indicators were performed by the bivariate Pearson correlation coefficient (r). Effect sizes (ES) were calculated similarly to the previous study [32]. The changes percentage was calculated by formula: $CP\% = \frac{{\left( {posttest - pretest} \right)}}{pretest} \times 100$. The significance level was set at *p* ≤ 0.05 for all statistical analyses.

## Results

### Physical and Psychological status

The descriptive data of performance and psychological variables are presented in Table 2. There were no significant differences between groups in all indices at baseline (*p* > 0.05). Although there was a substantial decrease in body mass in both groups who performed yoga, there were no significant differences between groups (F = 2.9, *p* < 0.070, ηp^2^ = 0.19). A 12-week intervention significantly decreased body fat percentage (BF%) in the Y-HVD and Y-LVD groups compared to the HVD group with a moderate effect size (F = 7.2, *p* < 0.003, ηp^2^ = 0.36). Handgrip strength was measured as a physical indicator. We observed a significant difference between groups at handgrip strength (F = 8.9, *p* = 0.001, ηp^2^ = 0.41) with a moderate effect size. However, yoga is not considered a strength-enhancing exercise. The Bonferroni post hoc test showed the handgrip strength test was significantly increased in both yoga groups than in other groups (*p* < 0.05). In addition, we observed significant differences in the QoL questionnaire between groups at global health (F = 15.0, *p* < 0.001, ηp^2^ = 0.54), functional scales (F = 12.9, *p* < 0.001, ηp^2^ = 0.49), and symptoms scales (F = 13.0, *p* < 0.001, ηp^2^ = 0.50) with a moderate effect size. Three months of yoga classes effectively improved the QoL in both the Y-HVD and Y-LVD groups compared to the HVD group (Table 2).

**Table 2:** Performance and psychological indicators of participants before and after performing the intervention

Variable	Group	Pre	Post	% change	P within	P between
Body mass (kg)	HVD	73.7 ± 12.7	73.5 ± 12.5	−0.11	0.727	0.070
	Y-LVD	68.1 ± 11.1	67.2 ± 10.7	−1.31	0.030	
	Y-HVD	74.5 ± 10.0	73.8 ± 9.7	−1.71	0.012	
Body fat percentage (%)	HVD	37.0 ± 4.4	36.8 ± 4.3	−0.52	0.343	0.003
	Y-LVD	37.0 ± 4.1	35.3 ± 4.3	−4.67*	0.001	
	Y-HVD	34.8 ± 3.3	33.4 ± 2.9	−3.94*	0.003	
Handgrip strength tests (kg)	HVD	18.0 ± 4.3	18.3 ± 4.3	1.73	0.345	0.001
	Y-LVD	16.4 ± 2.8	18.4 ± 2.4	13.32*	0.001	
	Y-HVD	18.7 ± 4.0	20.0 ± 4.3	7.09*	0.001	
Vitamin D (IU)	HD	41.2 ± 16.2	53.5 ± 15.9	34.62	0.001	0.005
	Y + HD	44.8 ± 13.1	57.5 ± 12.3	32.17#	0.001	
	Y + LD	43.4 ± 15.1	49.3 ± 16.2	15.69*	0.001	
**Quality of life questionnaire**						
Global health	HVD	70.8 ± 21.2	69.2 ± 22.2	−3.01	0.161	0.001
	Y-LVD	58.3 ± 14.7	75.8 ± 15.4	33.78*	0.001	
	Y-HVD	59.9 ± 17.5	82.5 ± 12.7	47.01*	0.001	
Functional scales	HVD	68.4 ± 18.2	70.7 ± 19.5	3.38	0.351	0.001
	Y-LVD	58.4 ± 19.8	81.1 ± 9.8	49.9*	0.001	
	Y-HVD	55.4 ± 17.9	84.2 ± 7.9	62.8*	0.001	
Symptom scales	HVD	34.8 ± 13.7	34.5 ± 12.6	3.85	0.928	0.001
	Y-LVD	41.8 ± 18.8	26.2 ± 11.6	−35.0*	0.001	
	Y-HVD	43.4 ± 12.2	18.6 ± 4.8	−54.1*	0.005	

*HVD* high dose of vitamin D, *Y-LVD* yoga with a low dose of vitamin D, *Y-HVD* yoga with a high dose of vitamin D.

*Significant difference with HVD group.

^#^Significant difference with Y-LVD group

### Vitamin D and cytokines levels

A 12-week VD supplementation period led to a significant increase in VD concentrations (F = 6.5, *p* = 0.005, ηp^2^ = 0.33). The post hoc analysis showed a significant difference was between both HD and Y + HD groups and the Y + LD group.

**Fig. 2:**
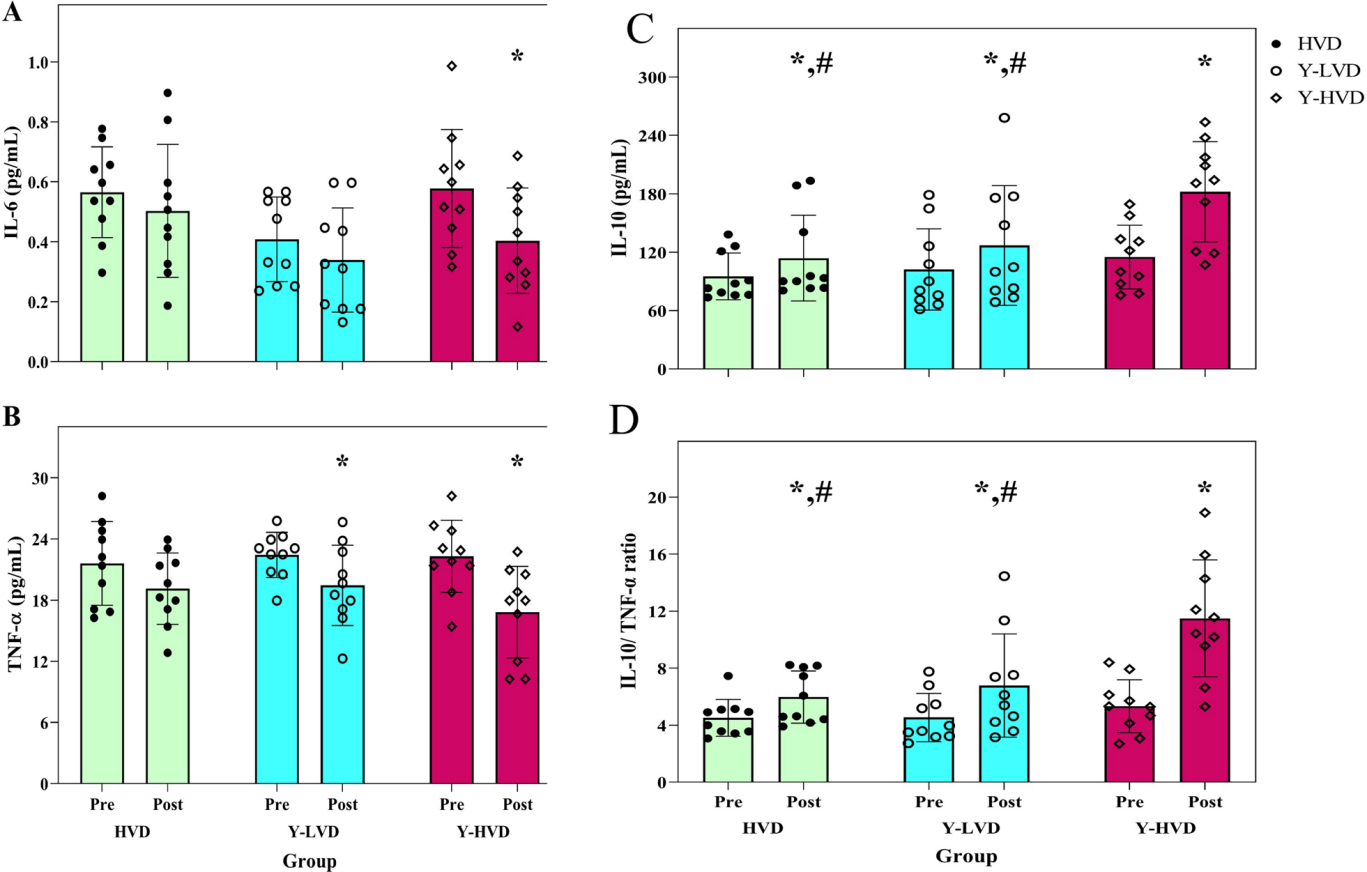
Concentration of serum IL-6 (**a**), TNF-α (**b**), IL-10 (**c**), and IL-10/TNF-α ratio (**d**) at baseline and after the intervention. HVD: a high dose of vitamin D, Y-LVD: yoga with a low dose of vitamin D, Y-HVD: yoga with a high dose of vitamin D. *Significant difference from pre-to post-intervention; #significant difference with the Y-HVD group

There were no significant differences in the circulatory level of IL-6 between groups (F = 1.2, *p* = 0.318, ηp^2^ = 0.08). Also, in intra-group changes, there was a substantial decrease in the Y-HVD group (*p* = 0.001, −30.9%), but there were no significant changes in the Y-LVD (*p* = 0.150, −16.3% and HVD group (*p* = 0.390, −10.2%) (Fig. 2a).

Although there were significant decreases in the circulatory level of TNF-α in the Y-LVD (*p* = 0.034, −13.0%) and Y-HVD groups (*p* = 0.001, −24.7%), not in the HVD group (*p* = 0.149, −8.7%), these changes were not significant between groups (F = 1.6, *p* < 0.230, ηp^2^ = 0.11) (Fig. 2b).

There was a significant difference in the circulatory level of IL-10 between groups (F = 5.7, *p* < 0.009, ηp^2^ = 0.31) with a moderate effect size. The Bonferroni post hoc test showed the difference between the Y-HVD group and the other groups (*p* < 0.05). Serum concentration of IL10 significantly increased in the Y-HVD (*p* = 0.001, 61.9%), the Y-LVD (*p* = 0.023, 19.4%), and the HVD groups (*p* = 0.025, 17.0%) (Fig. 2c).

The ratio of IL-10/ TNF-α was measured as an anti-inflammatory index. There was a significant difference in the IL-10/ TNF-α ratio between groups (F = 9.3, *p* < 0.001, ηp^2^ = 0.42) with a moderate effect size. The Bonferroni post hoc test showed changes in the Y-HVD group differ significantly from the other groups (*p* < 0.05). In intra-group changes, there were significant increases in the Y-HVD group (*p* = 0.001, 117.4%), the HVD (*p* = 0.021, 36.6%) and Y-LVD (*p* = 0.030, 48.8%) groups (Fig. 2d).

Table 3 presents the correlations between circulatory markers and weight, BF%, strength, and QoL indicators. There were significant negative relations between IL-10 changes and changes in weight (r = −0.51), BF% (r = −0.38), and positive association with global health on of the QoL indicator (r = 0.52). TNF-α and IL-6 changes only demonstrated a significant positive correlation with BF% (Table 3). Significant negative correlations between IL-10/ TNF-α ratio changes and weight (r = −0.38) and BF% (r = −0.59) changes and significant positive global health changes(r = 0.42) were observed (Table 3).

**Table 3:** Correlation between changes in inflammatory markers and weight, body fat%, and quality of life indicator after the intervention

Variables	∆ Weight	∆ BFP	∆HGS	∆ GH	∆ FS	∆ SS
∆ IL-6	0.15	0.43*	−0.03	−0.24	0.04	0.17
∆ TNF-α	0.03	0.36*	−0.13	−0.13	−0.01	0.04
∆ IL-10	−0.51*	−0.38*	0.19	0.52*	0.34	−0.33
∆ IL-10/ TNF-α ratio	−0.38*	−0.59*	0.28	0.42*	0.17	−0.24

*BFP* body fat percentage, *HGS* handgrip strength, *GH* global health, *FS* functional scales, *SS* symptom scales

*Significant correlation (*p* < 0.05)

### Gene expression in PBMCs

**Fig. 3:**
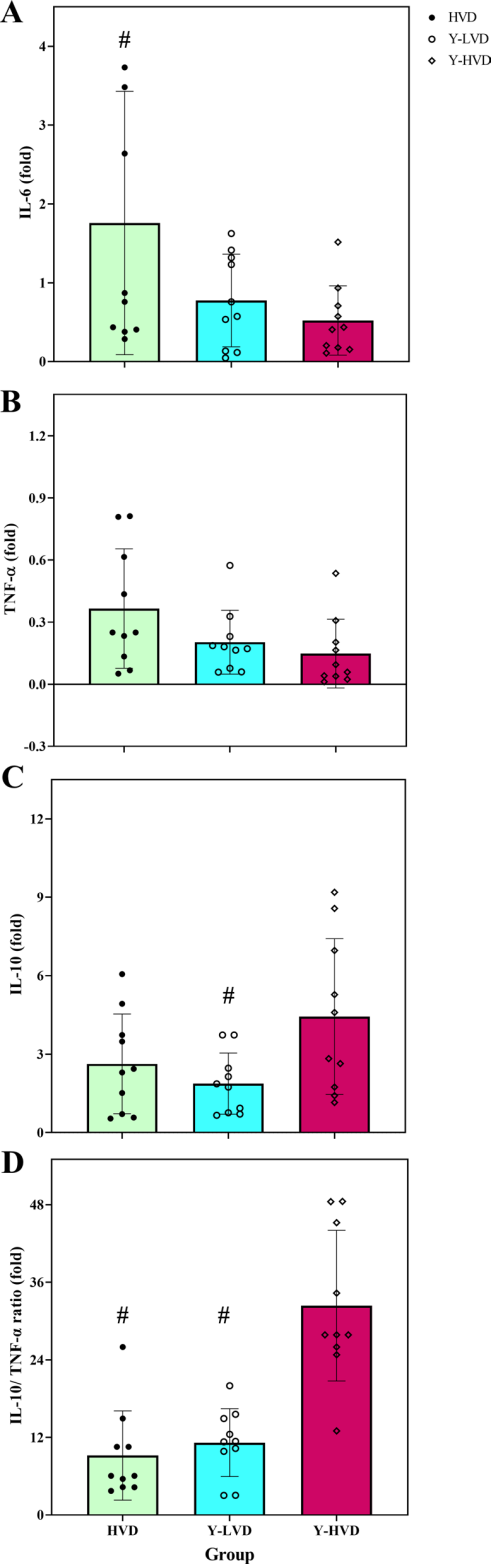
Gene expression of IL-6 (**a**), TNF-α (**b**), IL-10 (**c**), and IL-10/TNF-α ratio (**d**) at baseline and after intervention in peripheral blood cells. HVD: a high dose of vitamin D, Y-LVD: yoga with a low dose of vitamin D, Y-HVD: yoga with a high dose of vitamin D. *Significant difference from pre-to post-intervention; #significant difference with the Y-HVD group

Figure 2a shows the changes in IL-6 gene expression in the groups after intervention. A significant difference was observed in leukocyte's IL-6 expression between the groups (F = 3.8, *p* = 0.034). The Bonferroni post hoc test showed a significant difference was between the HVD group and Y-HVD. IL-6 expression upregulated in the HVD; doing yoga led to a decline in IL-6 expression (Fig. 3a).

Following the intervention, there were no significant differences in leukocyte's TNF-α expression between groups (F = 3.39, *p* = 0.075) (Fig. 3b).

In addition, a significant difference was observed in leukocyte's IL-10 expression between groups after intervention (F = 3.80, *p* = 0.036). Expression of IL-10 increased in all groups; the magnitude of the increase has significantly differed in the Y-HVD group and the Y-LVD group (Fig. 3c).

Interventions enhanced the anti-inflammatory index, IL-10/ TNF-α ratio, in the peripheral mononuclear cells. There were significant differences in the IL-10/ TNF-α ratio between groups (F = 23.5, *p* < 0.001). The Bonferroni post hoc test showed increases in the Y-HVD group (32.4%) differ significantly from the Y-LVD (11.2%) and the HVD (9.2%) groups (Fig. 3d).

## Discussion

The primary aim of this study was to evaluate the effectiveness of 12 weeks of yoga training combined with high VD does supplementation on cytokine profile and QoL in BC survivors. Also, possible relations between cytokine levels with functional and psychological indices were assessed. High VD dose supplementations led to significant increases in VD level than low VD dose. The findings indicate the high amount of VD alone did not significantly improve the systemic inflammation and QoL and performance indicators. Yet, substantial improvements in QoL, handgrip strength and body composition were observed in combination with yoga. Moreover, yoga with a high VD dose led to marked increases in the circulatory level of IL-10 and decreases in the concentrations of IL-6 and TNF-α. Also, the anti-inflammatory index was increased in the yoga plus high VD dose group. These results were relatively parallel with changes in inflammatory cytokines gene expression in peripheral blood cells. In addition, there were significant correlations between circulatory markers changes, especially IL-10 and IL-10/TNF-α ratio changes, and weight and BF% changes as well as global health of QoL indicators. Moreover, our findings indicated that a combination of yoga and a low dose of VD improved QoL, handgrip strength, and body composition but did not show synergistic effects on cytokine balance in genes expression levels in peripheral blood cells and plasma levels.

The findings supported previous research demonstrating that yoga improves BC survivors' QoL [35, 36]. In this regard, Vadiraja et al. [36], in a randomized controlled trial study, showed that six weeks of yoga significantly improved emotional function, cognitive function, and reduced adverse effects, which subsequently led to an improvement in the QoL of BC patients undergoing radiotherapy. Several mechanisms are proposed for improving the QoL with yoga, including promoting physical fitness and independence, improving social behaviors and the feeling of empathy resulting from group training, and reducing anxiety [37]. Interestingly, the improved QoL coincided with improving body composition and handgrip strength and boosting the immune system by lowering systemic inflammation.

Cancer-related fatigue and its treatments are associated with low activity resulting in adverse effects on body composition and muscle atrophy, leading to a decline in BC survivors' QoL. Elevated body mass via increased BF% could intensify sedentary behaviors and causes complications such as metabolic diseases. On the other hand, exercise interventions like yoga can increase energy expenditure as long as the energy intake is constant, leading to losing weight by burning fat and positively affecting body composition [38–41]. Our finding supported previous studies and showed that a 12-week yoga practice period reduced BF% by 4.7% and 3.9% in the Y-LVD and Y-HVD groups, respectively. In addition, decreased muscle strength is another complication of sedentary behaviors. It was reported that handgrip strength might be an important correlate of health in BC survivors and could be an adjuvant method for evaluation function abilities [42]. We observed a marked increase in the handgrip strength in both Y-HVD (7.1%) and Y-LVD (13.3%) groups. In this regard, substantial improvements in handgrip strength have been reported in patients with rheumatoid arthritis [43] and the affected side in BC patients [44] with yoga exercises. Yoga movements include stretching and muscular endurance exercises that can inherently enhance strength by promoting neuromuscular coordination, especially in those who have experienced extreme muscle weakness. Therefore, yoga as an exercise intervention is effective in lowering BF% and improving muscle strength.

Improved the QoL of the participants was associated with a reduction in systemic inflammation indicators and an improvement in the anti-inflammatory index. Following the intervention, the IL-6 gene expression in PBMCs increased in the HVD group, but yoga led to decreased it in both Y-LVD and Y-HVD groups; serum concentrations of IL-6 were reduced in all groups, but only it was significantly reduced in the Y-HVD group (30%). In this regard, Long parma et al. (2015) also stated that six months of yoga did not affect inflammatory serum markers such as IL-6, IL-8, and TNF-α [41]. In contrast, some researchers reported yoga could lower inflammatory markers, IL-6, and TNF-α [25, 45, 46]. However, little is known about mechanisms underlying reducing inflammatory markers with yoga, reducing BF%, and increasing in parasympathetic nervous system via the anti-inflammatory cholinergic pathway [47] lead to reduce inflammation. The observed significant positive correlation (r = 0.43) between IL-6 changes and BF% changes approved that reducing fat tissue with the combination of yoga with low and high VD doses was involved in decreasing IL-6 levels. In addition, given most BC survivors report problems with sleep [48] and sleep disturbance can activate inflammatory signaling, improve sleep quality with yoga might account for lowering inflammatory markers in BC survivors. On the other hand, VD can suppress the production of the pro-inflammatory cytokine, IL-6, through inhibition of p38 activation and cytokine production in leukocytes [49], as well as down-regulation of NF-κB expression in human lymphocytes [50]; thus, the greatest impact on reducing IL-6 was seen in the Y-HVD group. In addition, a systematic review proposed that possible effects of VD supplementation could induce auto-immunity effects with increasing regulatory T activity and suppressing Th17 responses [51].

Also, we observed a significant increase in the anti-inflammatory index, the IL-10/ TNF-α ratio, in the Y-HVD group compared to other groups following the intervention in the serum and gene expression levels. This change was due to a marked increase in an anti-inflammatory cytokine, IL-10, and a non-significant decrease in a pro-inflammatory cytokine, TNF-α. In this regard, researchers reported no significant changes in serum TNF-α levels after 10–12 weeks of yoga training [45, 46, 52] and a considerable increase in serum IL-10 levels after 12 weeks of yoga [53]. Researchers have attributed these improvements to the anti-inflammatory effects of exercise training [54] and to burning fat tissue [40]. Researchers reported that increased production of IL-10, an anti-inflammatory cytokine, leads to inhibition of the synthesis of pro-inflammatory cytokines, including TNF-α as well as its receptors [55, 56]. Also, the observed substantial correlations between IL-10 (r = −0.38) and TNF-α (r = 0.36) changes and BF% prove that a decrease in adipose tissue is involved at serum levels of these cytokines. Moreover, VD could suppress TNF-α production through inhibition of p38 activation [49] and down-regulation of NF-κB expression in human leukocytes [50] and regulate the IL-10 gene expression in human B cells, increasing serum IL-10 level [57]. In this study, a further increase in IL-10 gene expression in the HVD (2.6 fold) and Y-HVD (4.4 fold) groups compared to the Y-LVD group (1.8 fold) indicated that high VD dose has noticeable anti-inflammatory effects. On the other hand, it seems that the degree of adaptations is intensity-dependent. Given that moderate-intensity aerobic training led to a greater reduction in TNF-α levels than low-intensity exercise [58], it might need to perform yoga with higher intensity for a significant effect.

We acknowledge that there are some limitations to this study. Firstly, monitoring the participants' diets and determining the amount of VD intake from the diet was difficult because of the intervention duration. Secondly, individualizing the intensity of yoga in a group class is very difficult and complicated; however, we used the Borg scale, representing the degree of the mental and physical difficulty of the work done. Given that the intensity may be an essential factor in the concentration of inflammatory cytokines, we recommend that the effect of exercise intensity be investigated in future research. In addition, we recommend that the investigation be conducted over a longer length, and the participants would be followed up to determine the persistence of the adaptations.

## Conclusions

In conclusion, receiving high doses of VD is safe and effective in reducing inflammatory markers. Also, performing yoga training effectively improves the physical and mental status and QoL of BC survivors. Therefore, combining both approaches, yoga with a high VD dose, can be used as a practical approach to balance anti- and pro-inflammatory markers and promote physical and mental fitness in cancer survivors.

## Data Availability

Data would be available from the corresponding author on reasonable request.

## Abbreviations

BC: Breast cancer
QoL: Quality of life
IL-6: Interleukin-6
IL-10: Interleukin-10
TNF-α: Tumor necrosis factor-α
PBMCs: Peripheral blood mononuclear cells
VD: Vitamin D
HVD: High dose of VD
Y-HVD: Yoga with a high dose of VD
Y-LVD: Yoga with a low dose of VD
BF%: Body fat percentage
RPE: Rating of Perceived Exertion
ANCOVA: Analysis of covariance
ES: Effect sizes
